# Study on structured method of Chinese MRI report of nasopharyngeal carcinoma

**DOI:** 10.1186/s12911-021-01547-1

**Published:** 2021-07-30

**Authors:** Xin Huang, Hui Chen, Jing-Dong Yan

**Affiliations:** 1 School of Biomedical Engineering, Southern Medical University, Guangzhou, 510515 Guangdong China; 2 Shuguang Hospital, Shanghai University of Traditional Chinese Medicine, Shanghai, 201203 China; 3 Nanfang Hospital, Southern Medical University, Guangzhou, 510515 Guangdong China

**Keywords:** Structured medical text, Named entity recognition, Knowledge network

## Abstract

**Background:**

Image text is an important text data in the medical field at it can assist clinicians in making a diagnosis. However, due to the diversity of languages, most descriptions in the image text are unstructured data. The same medical phenomenon may also be described in various ways, such that it remains challenging to conduct text structure analysis. The aim of this research is to develop a feasible approach that can automatically convert nasopharyngeal cancer reports into structured text and build a knowledge network.

**Methods:**

In this work, we compare commonly used named entity recognition (NER) models, choose the optimal model as our triplet extraction model, and present a Chinese structuring algorithm. Finally, we visualize the results of the algorithm in the form of a knowledge network of nasopharyngeal cancer.

**Results:**

In NER, both accuracy and recall of the BERT-CRF model reached 99%. The structured extraction rate is 84.74%, and the accuracy is 89.39%. The architecture based on recurrent neural network does not rely on medical dictionaries or word segmentation tools and can realize triplet recognition.

**Conclusions:**

The BERT-CRF model has high performance in NER, and the triplet can reflect the content of the image report. This work can provide technical support for the construction of a nasopharyngeal cancer database.

## Background

With the development of information, medical records have shifted from text to digital. At present, most medical institutions in China have achieved information construction. With the advent of the big data era, medical data are showing explosive growth [1], and researchers are gradually realizing that medical data are crucial [2, 3]. Compared with paper reports, the digitization of image reports can reduce the workload of doctors, and the data are not easily lost and can be stored for a long time. However, image reports are essentially written by doctors, so it is difficult for computers to understand their meaning, thus leading to messy text classification. Although doctors have certain standards when writing medical records, there are also certain differences in the standards between institutions [4], which can lead to information islands. An urgent problem is to share data among different institutions. Therefore, determining how to efficiently use these data has become a challenge.

Nasopharyngeal carcinoma (NPC) is one of the most common cancers in southern China, and its morbidity and mortality are higher than those at the world level [5]. As a product of medical digital technology, electronic medical records (EMRs) detail the descriptions related to diseases and can be a knowledge base of diseases. EMRs are also one of the most important information carriers in the medical field. A study shows that the quality of structured reports (SRs) is higher than that of free-text reports [6]. SRs can even improve patient management and provide technical support for clinical decision making (CDM). An interesting finding is that doctors prefer SRs to free-text reports [7–9]. The application of SRs in ultrasound imaging [10], computed tomography (CT) [11], and magnetic resonance imaging (MRI) [12] shows that SRs have great potential in clinical research. Therefore, the popularization of EMRs is conducive to medical data sharing. The disease knowledge network constructed based on structured data is of great significance to the research of NPC.

The early application of text structuring lies in English texts. The main steps of structuring are entity recognition and entity relationship matching. In other words, it extracts the concept of < entity, attribute, value > in the text to form structured information. The MedLEE system developed by Friedman et al. [13] can automatically map the entire clinical document to the modifier code. Specifically, the method extracts information from the clinical narrative text and uses the Unified Medical Language System (UMLS) to represent the extracted structured information. Denecke [14] combined existing language engineering methods and semantic conversion rules to map grammatical information to semantic roles and map chest X-ray reports to semantic structures. The system achieves 80% accuracy in detecting medical narratives. With the increasing maturity of English natural language processing technology, Skeppstedt et al. [15] attempted to apply the NER method to Swedish health records and evaluate its performance. They used the Conditional Random Field (CRF) model to identify four entities from clinical documents and found that the Sweden NER results are in line with English text.

Since Chinese is different from English, there is no space to separate words. If a single Chinese character is used for encoding, it does not seem to conform to the law. Therefore, it is necessary for Chinese word segmentation in preprocessing. However, the commonly used segmentation tools (e.g., LTP of Harbin Institute of Technology [16], ICTCLAS of Chinese Academy of Sciences [17], FNLP of Fudan University [18], etc.) are all trained based on daily corpus or news corpus. Some medical terminologies cannot be recognized well, so researchers usually construct medical dictionaries to solve this problem. On the basis of word segmentation tools, Shang et al. [19] used word co-occurrence frequency to find new words and build a medical dictionary, which greatly improved the accuracy of word segmentation. Chen et al. [20] proposed a method for structured processing of microscopic text data based on statistical information. They constructed a medical dictionary through statistical methods after the text clustering. The obtained structured data do not rely on word segmentation tools. Tian et al. [21] used cosine similarity to merge ambiguous terms. They extracted information and generated structured templates through dependency syntax analysis. The values of accuracy in the extraction of attribution and value are 82.91% and 79.11%, respectively.

In this study, we developed a named entity recognition (NER) model based directly on triplet and expanded it. The model can recognize named entities at the character level, which eliminates the need for word segmentation. This capability is significantly different from existing structuring approaches, as our method can reduce the cumulative error caused by multi-level tasks. The contributions of our work are summarized as follows:

1. After evaluating the MRI report of NPC, we found that a large number of descriptions consist of subject, predicate, and object. This structure is similar to triplet. Thus, we designed a new triplet architecture by extending the subsidiary entity and location to better cover all sentences.
2. The NER algorithm uses the BERT-CRF architecture. We compared several commonly used models, tested the performance of the model, and optimized the model in a manually annotated data set.
3. This study advances the application of a rule base in the field of triplet relation extraction. This method only applies a few rules to achieve good performance. Combined with BERT-CRF model, its extraction rate can reach more than 80%.
4. Finally, we presented the structured results through the knowledge network, which clearly reflects the imaging findings of NPC and may help clinical diagnosis and differentiation.

The remainder of this paper is organized as follows. Section 2 proposes a novel triplet and a NER model based on the triplet. Section 3 describes the methodology for data analysis and experimental results. Section 4 summarizes the strengths and weaknesses of the experiments, and looked forward to future work. Finally, Sect. 5 presents conclusions and implications.

## Methods

At present, the challenge of text structuring is the complexity of semantics. How to make the computer understand the meaning of medical text is the key to solving the problem. Text structure can be divided into pre-structured and post-structured according to different methods. The former defines some structured rules before writing medical records. Clinicians choose the corresponding terminology to write the report based on the images. The report is structured when finished, but due to the limitation of the rules, it cannot cover all image descriptions. This deficiency results in deviations between images and textual information. Therefore, structuring often adopts post-structuring methods.

Most methods are based on the medical dictionary or word segmentation tools. Statistical information, such as word frequency, is also used to extract structured content. These statistical characteristics are closely related to the amount of text. However, medical reports are usually written in abbreviations and non-standardized terms [22], making building a comprehensive dictionary a difficult problem. Aiming at the characteristics of the examination reports of NPC, this work uses neural network-based methods to directly identify the triplet in the text. It does not rely on dictionary or word segmentation tools to reduce the error caused by a superior task. Finally, we construct a knowledge network of NPC based on the structured results, which can provide an objective data basis for clinical research.

**Fig.1:**
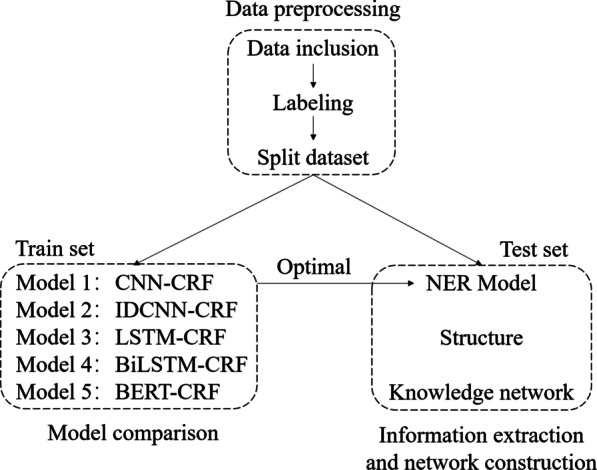
Overall framework. The overall framework can be divided into three parts: data preprocessing, NER model training, Information extraction and network construction

In view of the characteristics of the MRI report of NPC, we propose a new method for constructing a knowledge network. As shown in Fig. 1, we formulate a ternary composition based on the report content. After data preprocessing, the text is labeled with ternary components and then the information extraction model is obtained through neural network training. The model then determines whether the ternary component meets the requirements according to the rule base and finally visualizes the extracted structured information to construct a knowledge network.

### Triplet architecture

To realize the structuring of the NPC reports, we propose a new triplet architecture based on the original < entity, attribute, value > . To better summarize the text content, the entity expands into two parts, namely, < (Primary entity, Subsidiary entity), Attribute, Value > . Primary entity (**P**) is a certain part of the human body, such as the nasopharyngeal cavity, ethmoid sinus, and sphenoid sinus. Subsidiary entity (**S**) is the supplementary part of **P**. Attribute (**A**) is the description of the physical characteristics, such as diameter, CT value, and flat scan signal. Value (**V**) is the description result of the entity or attribute, such as the nasopharyngeal cavity (asymmetric), diameter is (3 cm), and plain scan signal is (high/low). In the description of sentences, there are often words like “seeing” and “existing.” Deleting these words does not affect the structured process. As shown in Table 1, we divide the sentence into four categories of PSAV according to the vocabulary, which is mediated by clinicians.

**Table 1:** Example of triplet

Sentence	The scan showed that the nasopharyngeal cavity was asymmetry and severely narrowed, tumor size was seen in 1.2 cm × 0.9 cm			
PSAV vocabulary	Primary entity	Subsidiary entity	Attribute	Value
	Nasopharyngeal cavity	Tumor	Size	Asymmetry, severely narrowed, 1.2 cm × 0.9 cm

### Text structuring based on named entity recognition

After obtaining the PSAV vocabulary, we mark the data according to the vocabulary using the BISO tagging method [23]. B represents the beginning position of the word, I represents the inside and end of the word, S represents a single entity, and O means that the word does not belong to any entity. If the word belongs to the primary entity, then “-P” will be added after BISO to indicate the ternary composition of the word. For instance, entity “nasopharyngeal cavity” belongs to the primary entity, and so its label is “nasopharyngeal B-P, cavity I-P.” This labeling method can not only judge whether a word is a ternary component but also accurately define the boundary of the word. Different from the previous structured methods, we do not segment all the contents of the document but only identify and label the words belonging to the vocabulary. Words that do not belong to the vocabulary are marked as O.

This study uses a neural network-based NER model to identify the < (P,S),A,V > . Deep neural networks can effectively extract features of text and images [24, 25]. The NER model is mainly composed of two parts: context encoder architecture and tag decoder. Commonly used context encoder architectures are CNN networks (CNN, IDCNN [26], etc.), RNN networks (LSTM [27], GRU [28], BiLSTM [29], etc.), and pre-trained language models (BERT [30], GPT [31], ELMo [32], etc.). The architecture can effectively deal with the one-dimensional sequence, such as text, voice and ECG [33, 34]. First, we initialize a trainable look-up matrix $C^{N \times M}$ (where $N$ represents the size of the vocabulary, and $M$ represents the dimension of the embedding word vector). Each word is converted into an index so the row vector $w_{index}$ of the look-up matrix represents the M-dimensional dense vector of the word. The low-dimensional vector obtained by the look-up matrix is used as the input of the neural network; after operations such as convolution and linear transformation, the network outputs a vector with semantic information. Finally, the tag decoder decodes the outputs of the context encoder to obtain the tag of each word in the sentence. The commonly used decoder is fully connected layer + CRF.

CRF was proposed by Lafferty et al. [35]. In the CRF, the value of the current position is only related to its adjacent positions. Let $X = (X_{1} ,X_{2} ,X_{3} , \ldots ,X_{n} )$ be the text sequence, and $Y = (Y_{1} ,Y_{2} ,Y_{3} , \ldots ,Y_{n} )$ be the entity label of the sequence. Given a text sequence $X$, the probability distribution $P(Y|X)$ of the entity category of the text is called CRF.

In CRF, we need to define two functions. The first function is called node feature, which is only related to the current node and is denoted as

1 $$s_{l} (y_{i} ,x,i),\,\,\,\,\,l = 1,2, \ldots ,L$$

where *L* is the total number of node feature functions defined at the node, and *i* is the position of the current node in the sequence. The second function is called local feature, which is related to the current node and the previous node and is denoted as

2 $$t_{k} (y_{i - 1} ,y_{i} ,x,i),\,\,\,\,k = 1,2, \ldots ,K$$

where $K$ is the total number of local feature functions defined at the node. Whether it is the node feature function ${s}_{l}$ or the local feature function ${t}_{k}$, their values are 0 or 1. A weight λ and μ are respectively assigned to the feature function. The complete linear CRF is expressed as

3 $$P(y|x) = \frac{1}{Z(x)}\exp \left\{ {\sum\limits_{i,k} {\lambda_{k} t_{k} (y_{i - 1} ,y_{i} ,x,i) + \sum\limits_{i,l} {\mu_{l} s_{l} (y_{i} ,x,i)} } } \right\}$$

where Z(x) is the normalization factor.

4 $$Z(x) = \sum\limits_{y} {\exp \left\{ {\sum\limits_{i,k} {\lambda_{k} t_{k} (y_{i - 1} ,y_{i} ,x,i) + \sum\limits_{i,l} {\mu_{l} s_{l} (y_{i} ,x,i)} } } \right\}}$$

The CRF takes into account the state of the previous moment, making the predicted sequence label more standardized, and there will be no case where the previous word label is Begin and the next word label is still Begin (just let $t(y_{i - 1} = Begin,y_{i} = Begin,x,i) = 0$). Therefore, constructing the feature functions is the key in the process of solving the CRF.

In this study, we compare five models (CNN-CRF, IDCNN-CRF, LSTM-CRF, BiLSTM-CRF, BERT-CRF) and choose the optimal model as the NER model. Given that the description of an image report is relatively long, the obtained results are slightly lower than those for the same model on short sentences. Therefore, we divide the sentence into tokens according to the comma and period. The labeled data are used to train the model and select the best one. Then, the PSAV component of the sentence is output by Algorithm 1. In the process of structuring, we find that its ternary component is not strictly consistent, such as the Chinese characters “和,” “及,” and “并,” they all mean “and” in English. Therefore, we labeled these characters as C (Conjunction) and constructed a conjunction rule database (Table 2).

**Table 2:** Example of conjunction rule base

Entity labels	Tokens	Triplet < (P,S),A,V >
PCP	No abnormalities/V in ethmoid sinus/P and/C maxillary sinus/P	< (Ethmoid sinus,–),–, no abnormalities >
		< (Maxillary sinus,–),–, no abnormalities >
ACA	No change/V in thyroid shape/A and/C signal/A	< (Thyroid,–),shape, no change >
		< (Thyroid,–),signal, no change >
VCV	Nasopharyngeal cavity/P Asymmetry/V with/C mild stenosis/V	< ( Nasopharyngeal cavity,–),–, > Asymmetry, mild stenosis


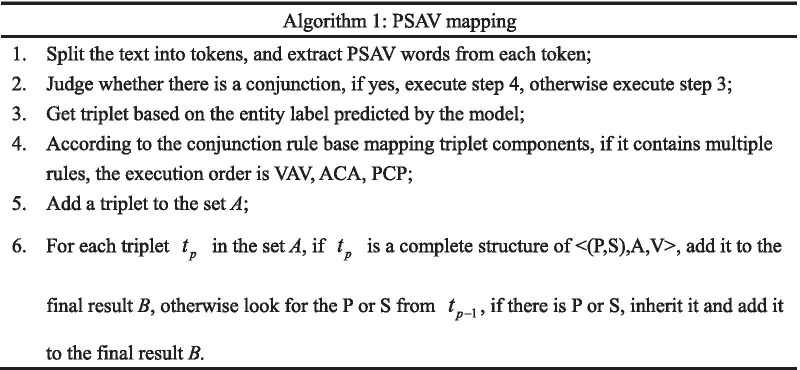


According to the conjunction rule database, we structure the sentences with conjunctions into multiple ternary groups to ensure that the relationship between entities and other components can be a one-to-one correspondence. A complete triplet contains at least an entity (P or S) and a value V. Since we divide the text with comma or period, some tokens will lack entities but can still be found in the previous token. Taking Table 1 as an example, the token “severe stenosis” contains only the value. However, the previous token contains the primary entity “nasopharyngeal cavity,” and “severe stenosis” is the value of “nasopharyngeal cavity,” and so it inherits the primary entity of the previous token to form a complete triplet.

### Knowledge network

**Fig. 2:**
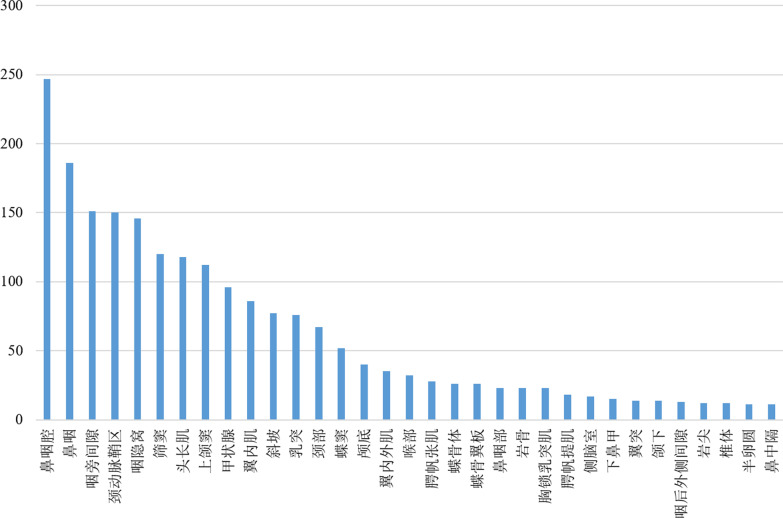
Word frequency of the primary entities. The entities from left to right are: nasopharyngeal cavity, nasopharyngeal, parapharyngeal space, carotid sheath, pharyngeal crypts, ethmoid sinus, musculus longus capitis, maxillary sinus, thyroid, pterygoid muscle, clivus, mastoid, neck, sphenoid sinus, skull base, internal and external pterygoid muscles, throat, tensor veli palatine, sphenoid body, sphenoid wing plate, nasopharynx, petrous bone, sternocleidomastoid muscle, levator veli palatine, lateral ventricle, inferior turbinate, pterygoid process, submandibular, posterolateral pharyngeal space, petrous apex, vertebral body, semiovale, nasal septum

In the last step, the acquired triplets are visualized as an NPC knowledge network. Structured information is a path of the network. Therefore, there will be four nodes in a path representing the triplet architecture < (P,S),A,V > . We use an empty circle to represent the NONE. The initial node is the primary entity, and the size of the network depends on the number of primary entities. We count the number of sentences containing the primary entities and screened out the primary entities with a word frequency greater than or equal to 20 (Fig. 2). The descriptions of these entities can cover most of the imaging features of NPC. In this way, the knowledge network is universal. An MRI scan of the nasopharynx will usually include the head and neck, but we only focus on the physical words of the nasopharynx. The primary entities of the nasopharynx selected from Fig. 2 are: “鼻咽, 咽旁间隙, 咽隐窝, 头长肌, 翼内外肌, 斜坡, 腭帆张肌, 蝶骨体, 蝶骨翼板, 腭帆提肌, 翼突” (In English: nasopharyngeal, parapharyngeal space, pharyngeal recesses, longus capitis, internal and external pterygoid muscles, clivus, tensor veterinus, sphenoid body, sphenoid wing plate, levator veterinus, pterygoid process).

### Experimental details

In the part of the NER model, we set some hyperparameters to adjust the performance of the model. As the model can only handle fixed-length sequences, we divide the text into tokens p and padding to the max sequence length = 50. We use Adam optimizer (learning rate is 0.001) to train the model for 50 epochs. In the training process, the batch size is 100, and the loss function is cross entropy. The shape of the look-up matrix C is 579 × 50. In particular, the epoch = 10 and batch size = 8 for the BERT-CRF model.

## Result

### Data set and preprocessing

The experimental data come from the MRI report of NPC in a large tertiary hospital and have all been desensitized. The patient ID, name, and examination number were deleted, and only the “description” field was retained. Finally, 769 samples were collected and divided into 6:2:2, of which 461 samples were used to train NER models, 154 samples were used to verify and select the optimal model, and another 154 samples were used to structure and build knowledge networks. The data used for training and verification were labeled with triplet. In addition, the description of the nasopharynx mostly included words such as “left side wall” and “parietal posterior wall.” Hence, we label these words as **L** (Location).

### Comparison of named entity recognition models

The model evaluation adopts accuracy rate and recall rate. In NER, the metrics are usually calculated on the basis of the entity level rather than a single word. Accuracy rate indicates the number of correct entities among the predicted entities. Recall rate indicates the number of correct predictions in the sample total entities.

**Fig. 3:**
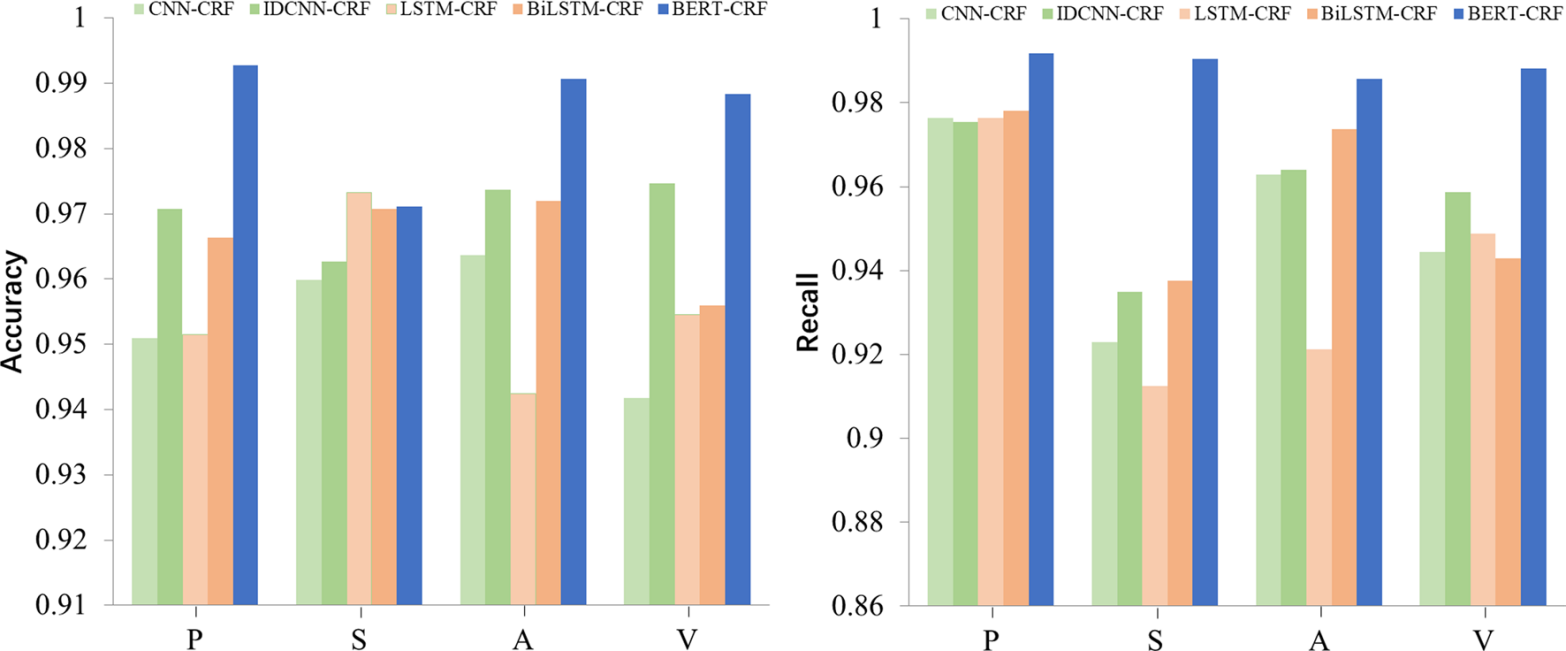
Performance of NER model. The accuracy(left) and recall(right) of the NER model. The BERT-CRF performs best. In the CNN-Architecture, the performance of IDCNN-CRF is better than CNN-CRF, while BiLSTM-CRF is better than LSTM-CRF in the RNN-Architecture

The comparison of NER models is shown in Fig. 3. The left picture shows the accuracy of the model, and the right picture shows the recall rate. The figure illustrates that the performance of the BERT-CRF model is better than those of the other four models. We likewise found that the model has lower accuracy and recall rate for the subsidiary entity, probably because the subsidiary entity is relatively small in the four categories, accounting for only 16.12%. However, the BERT-CRF model has relatively flat fluctuations in triplet. Therefore, we choose the BERT-CRF model as the information extraction model for this experiment.

### Analysis of structured results

After obtaining the trained information extraction model, we use Algorithm 1 to structure 154 samples of text data. In addition, only when label L is adjacent to the primary entity will it be added to the structured table. Table 3 is an example of the text structure, and from Table 4, we can see that if the sentence token contains only one set of triplet, then the algorithm can well identify the corresponding triplet. However, the structure of the compound sentences is not very precise, which is also a defect of this algorithm. Without considering the label L, the structured information extraction rate of the text is 84.74%, and the accuracy rate of the structure is 89.39%.

**Table 3:** Example of structure

Sentence	The nasopharyngeal cavity is slightly asymmetrical and slightly narrow. The posterior wall and bilateral walls of the nasopharynx are thickened. A lump formed on the posterior wall of the nasopharynx. The bilateral pharyngeal recesses are narrowed. The T1WI of the lump shows a uniform signal, and theT2WI becomes a high signal					
Location	Primary entity		Location	Subsidiary entity	Attribute	Value
–	Nasopharyngeal cavity		–	–	–	Asymmetrical
–	Nasopharyngeal cavity		–	–	–	Slightly narrow
–	Nasopharynx		Posterior wall	–	–	Thickened
–	Nasopharynx		Bilateral walls	–	–	Thickened
–	–		Posterior wall	Lump	–	Formed
Bilateral	Pharyngeal		–	–	–	Narrowed
–	–		–	Lump	T1WI	Uniform signal
–	–		–	Lump	T2WI	High signal

**Table 4:** Limitation of structured algorithm

Description	Triplet < (P,S),A,V >	Notes
There exists multiple lymph nodes in the parapharyngeal space, the size of which is 2.6 × 1.5 cm	< ( Parapharyngeal space,–), lymph nodes, multiple >	After dividing the description by punctuation, each token has only one triplet
	< (–, Lymph nodes),size, 2.6 × 1.5 cm >	
There exists multiple lymph nodes with the size of 2.6 × 1.5 cm in the parapharyngeal space	< ( Parapharyngeal space, lymph nodes),size, common >	This description has two triples

### Knowledge network of nasopharyngeal cancer

**Fig.4:**
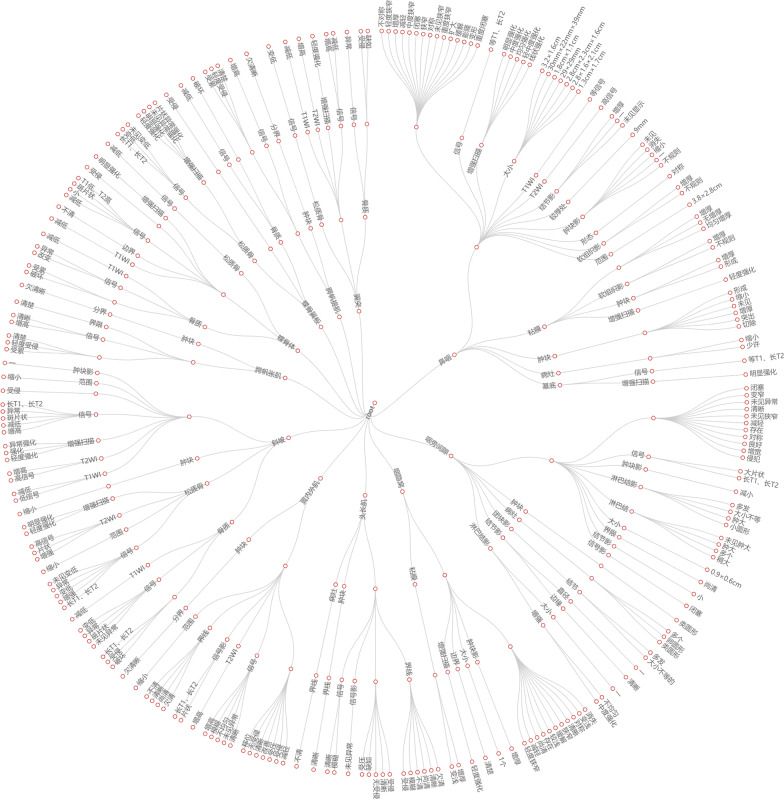
Knowledge network of nasopharyngeal cancer

After getting the structured information in the previous section, we filter out the structured table containing nasopharyngeal vocabulary according to Fig. 2. Considering that some words in the structured table have the same meaning (e.g., normal, no abnormality, etc.), we merge these words into the same description. The final knowledge network is shown in Fig. 4.

## Discussion

The structured method proposed in this study does not rely on medical dictionaries and word segment tools. We obtain the structured content of the text through the neural network model and build a knowledge network of nasopharyngeal cancer. In the conventional method, the text sequence is segmented into words. Afterwards, the statistical features, such as word frequency, parts of words, words similarity, and so on, are artificially constructed. Researchers use these features to structure the text and get the triplet. However, these feature values are different in data sets of different sizes. When the data set is large, the features will tend toward a stable distribution. In our method, we build an end-to-end structured model of Chinese MRI reports. This model can automatically mine semantic features and has good performance on small-scale data sets. It provides a new feasible solution for the structuring of image reports and technical support for text processing, such as text classification and information retrieval. Finally, we visualize the knowledge network, which could provide us with more valuable information about nasopharyngeal cancer. However, our work still has the following problems:

1. The named entity model has higher requirements for hardware. The parameters of the BERT model are 340 M, even the base version model has 110 M parameters, which is costly for model deployment and use.
2. The current structure algorithms are insufficient for the long sentences and compound sentences.
3. Our research only collects data from one medical institution, and the general applicability of the model and algorithm has not been explored. Different clinicians will have slightly different descriptions of the same phenomenon as well, and these terms need to be merged and normalized manually.

Therefore, in the follow-up work, more sources of data will be collected to improve the applicability of the model. At the same time, we will focus on the structure of compound sentences and the normalized description of the same medical phenomenon as well as continuously optimize model parameters and algorithms. With the expectation that it will be applied to the image report data of more diseases, a comprehensive knowledge network of multiple diseases is established to provide reliable and effective support for CDM and scientific research analysis.

## Conclusion

The BERT-CRF model can effectively extract the entities in the MRI report, which provides a convenient technology for the structuring of medical texts. At the same time, we can intuitively know the clinical characteristics of NPC through the knowledge network.

## Data Availability

All data and codes are available from Github (https://github.com/saynHuang/npc_structure).

## Abbreviations

NPC: Nasopharyngeal carcinoma
EMR: Electronic medical records
CDM: Clinical decision-making
SRs: Structured reports
CT: Computed tomography
MRI: Magnetic resonance imaging
UMLS: Unified medical language system
NER: Named entity recognition
CRF: Conditional random field
PSAV: Primary entity, subsidiary entity, attribute, value
